# Dissociable Neural Mechanisms for Human Inference Processing Predicted by Static and Contextual Language Models

**DOI:** 10.1162/nol_a_00090

**Published:** 2024-04-01

**Authors:** Takahisa Uchida, Nicolas Lair, Hiroshi Ishiguro, Peter Ford Dominey

**Affiliations:** Ishiguro Lab, Graduate School of Engineering Science, Osaka University, Osaka, Japan; INSERM UMR1093-CAPS, Université Bourgogne Franche-Comté, UFR des Sciences du Sport, Dijon, France; Robot Cognition Laboratory, Marey Institute, Dijon, France

**Keywords:** automatic, explicit, implicit, inference, language model, minimalist, strategic

## Abstract

Language models (LMs) continue to reveal non-trivial relations to human language performance and the underlying neurophysiology. Recent research has characterized how word embeddings from an LM can be used to generate integrated discourse representations in order to perform inference on events. The current research investigates how such event knowledge may be coded in distinct manners in different classes of LMs and how this maps onto different forms of human inference processing. To do so, we investigate inference on events using two well-documented human experimental protocols from Metusalem et al. (2012) and McKoon and Ratcliff (1986), compared with two protocols for simpler semantic processing. Interestingly, this reveals a dissociation in the relation between local semantics versus event-inference depending on the LM. In a series of experiments, we observed that for the static LMs (word2vec/GloVe), there was a clear dissociation in the relation between semantics and inference for the two inference tasks. In contrast, for the contextual LMs (BERT/RoBERTa), we observed a correlation between semantic and inference processing for both inference tasks. The experimental results suggest that inference as measured by Metusalem and McKoon rely on dissociable processes. While the static models are able to perform Metusalem inference, only the contextual models succeed in McKoon inference. Interestingly, these dissociable processes may be linked to well-characterized automatic versus strategic inference processes in the psychological literature. This allows us to make predictions about dissociable neurophysiological markers that should be found during human inference processing with these tasks.

## INTRODUCTION

We are witnessing an interesting conjuncture in the science and technology of language. Language models (LMs) in machine learning are beginning to display remarkable performance capacities, with human-like performance in question answering (Talmor et al., 2019), semantic similarity judgment, translation, and other domains (Devlin et al., 2019; Reimers & Gurevych, 2019). In certain aspects they are similar enough to human performance that specific measures of human language comprehension from psycholinguistic experiments are now being used to characterize and evaluate these LMs (Ettinger, 2020; Ettinger et al., 2016). At the same time, LMs are beginning to display underlying representations and mechanisms that provide insight into human brain processes in language processing (Dehghani et al., 2017; Mitchell et al., 2008; Schrimpf et al., 2021).

A central requirement in language comprehension is inference, the ability to complete missing information to allow disambiguation of meaning, as characterized in the tradition of research demonstrating that discourse comprehension often involves instantiation of relevant, unstated information accessed from long-term memory (Graesser et al., 1994; McKoon & Ratcliff, 1986, 1992). Metusalem et al. (2012) demonstrated that humans discriminate expected versus unexpected words based on the discourse context that requires causal or bridging inferences that engage general event knowledge that is not explicitly stated in the discourse. For example, knowledge about car accident events allows one to expect that if a car comes racing though a red light, a crash is imminent.

McKoon and Ratcliff (1992) made the distinction between prevalent constructivist theories of inference. They proposed that readers do not automatically construct inferences to fully represent the situation described by a text. Rather, in the absence of specific, goal-directed strategic processes, only inferences that are locally coherent and that rely on information that is quickly and easily available are processed. We refer to this as implicit inference. These minimal representations provide the basis for more strategic inference processes that readers invoke to achieve more specific comprehension goals (McKoon & Ratcliff, 1992; van den Broek et al., 2015). We refer to this as explicit inference.

The current research investigates how event knowledge may be coded in LMs developed in the context of natural language processing (NLP) in machine learning. We set out to examine how LMs may be able to demonstrate human-like performance in tasks that are thought to require knowledge of general events that is not directly available in the discourse being processed. This has recently been demonstrated as wikipedia2vec, a word2vec LM that learns to predict the current word based on the surrounding context words, trained on the entire Wikipedia corpus (Yamada et al., 2020), was used to predict brain responses during discourse processing that requires inference on events (Uchida et al., 2021). The human-like performance of the model was characterized by a specific linking hypothesis between the model behavior and human behavior and event-related potential (ERP) responses as in (Brouwer et al., 2017). As noted by Talmor et al. (2020), “if a pre-trained language model is based on word occurrence statistics, we would imagine that it would struggle on tasks that require symbolic reasoning, such as making inferences about predictable events. If a pre-trained model succeeds in such tasks, then the representations that it develops must be useful for these tasks.”

This motivates the current research, which explores in a more systematic way the properties that allow LMs to display human-like performance in these inferencing tasks. Metusalem et al. (2012) set out to address “how activation of particular elements of event knowledge is constrained in real time by the specific cues present in an unfolding sentential context.” We have a similar objective, which is to address this same question in the context of discourse processing by artificial LMs.

We will exploit the ability of current LMs to generate embeddings which can be used to resolve inference problems. Following Goldstein et al. (2021) we consider two types of LMs and their corresponding word embeddings: static embeddings (e.g., word2vec (Mikolov et al., 2013) and GloVe (Pennington et al., 2014)), which assign a single vector to each word in the lexicon irrespective of context, and contextual embeddings (e.g., BERT; Devlin et al., 2019), in which the same word is assigned different embeddings (vectors) as a function of the surrounding words. We test the hypothesis that these dissociable forms of LM will correspond to dissociable forms of inference processing. If our hypothesis bears out, then it will lead to predictions about dissociable neurophysiological signatures for these distinct inferencing processes.

Part of the originality of our approach is to examine potentially dissociable aspects of the human behavior called inference. In the following we attempt to establish a link between these processes and different classes of LMs, with the goal of then providing a mechanistic basis for making predictions about corresponding dissociable neural processes in the human brain.

## MATERIALS AND METHODS

We investigate inference on events using two well-documented protocols from Metusalem et al. (2012) and McKoon and Ratcliff (1986), in an effort to demonstrate dissociation in the relation between implicit versus explicit inference, depending on the LM.

In the Metusalem task, subjects are exposed to a sentence, or to the same sentence preceded by an event-evoking discourse. They are then tested on one of three types of words: Expected (expected both in the context of the sentence alone and in the extended discourse), Unexpected-Related (unexpected in the context of the sentence alone but related to the extended discourse), Unexpected-Unrelated (unexpected in the context of the sentence alone and unrelated to the extended discourse). The measure of performance is the N400 brain responses recorded by EEG scalp electrodes. We should note that there is no explicit or strategic task performed by the subject, and we thus consider this an implicit inference task.

We recall that the N400 is a cortically evoked brain potential that occurs ∼400 ms after a given word onset. Its amplitude reflects a form of semantic dissonance or surprise (Kutas & Hillyard, 1980), or the difficulty of understanding a word in a given context. An example from Metusalem of such a sentence, a discourse and the three types of words, are given in Table 1. In the sentence context, N400s are increased for both Unexpected types with respect to Expected. In the event-evoking discourse, the N400 for the Unexpected-Related type is rescued (i.e., the discordance response is reduced, revealing access to event knowledge that allows inference). That is, the N400 for the Unexpected-Related word is reduced, because the discourse context has made more explicit its relatedness. In the automatic versus strategic dissociation (McKoon & Ratcliff, 1992), this task would tend toward automatic inference processing.

**Table 1.** Example of stimuli and results in the Metusalem inference task

		Expected	Unexpected-Related	Unexpected-Unrelated
		**CRASH**	**POLICEMAN**	**CONDUCTOR**
		**N400 Size**		
Sentence alone	A moment later, she heard a terrible	Small	Large	Large
Discourse context + Sentence	Elizabeth was standing at the intersection waiting for the light to change. All of a sudden she saw a car barrel through the red light. A moment later, she heard a terrible	Small	Small-medium (N400 “rescued” by discourse)	Large

*Note*. Task based on Metusalem et al. (2012).

In McKoon’s inference task, subjects are exposed to one of two sentences that either evokes a context, for example, about writing a letter, or uses many of the same words but does not evoke that context. Subjects are then asked if a specific word, related to the context, appeared in the sentence. The experiment shows that subjects are slower to report that the target word (that can be inferred by the contextual but not control sentence) did not appear in the sentence only for the context-evoking sentences, revealing access to event representations that prime the target word. An example of two such sentences and a target word are given in Table 2. Given that the subjects have a strategic task to perform that requires inference processing, we consider that on the automatic versus strategic dissociation, this task tends toward strategic.

**Table 2.** Example of stimuli and results in the McKoon inference task

	Target word
	**WRITE**
Context Sentence: The debutante owed a letter to her mother, so she picked up some paper and sat down.	Slower response
Control Sentence: Like her mother, the debutante lettered in sports and often had her name in the paper.	Faster response

*Note*. Task based on McKoon and Ratcliff (1986).

We previously reproduced the Metusalem results using a model that constructed a discourse vector as a form of average of Wikipedia2Vec embeddings (Uchida et al., 2021), implemented numerically or approximated by a recurrent reservoir network. In the current research, we compare inference performance and semantic performance for the static and contextual models, respectively, based on word2vec/GloVe and BERT. BERT is designed to encode sentence context, and we thus predicted that it would demonstrate an inference processing capability that can be dissociated from that of the static LMs.

The objective of this study is thus to evaluate these static versus contextual LMs in different semantic and inference tasks, in order to identify dissociable computational processes, which can then be used to generate predictions about corresponding dissociations in neurophysiological processing when humans perform these same tasks.

### NLP Models and Simulation

In order to broadly evaluate the two classes of LMs, and in order to have sufficient data points to perform correlation analyses, we studied a number of instances of static and contextual models. The different instances of static models were derived from word2vec and GloVe models trained on different corpora, with different embedding vector dimensions. Similarly, the different instances of contextual models were derived from variants of BERT and RoBERTa in the Sentence-BERT context, which were pretrained and fine-tuned on various corpora as indicated in Table 1. While the static and contextual models thus varied in the pretraining and fine-tuning they were exposed to, the principal distinction remained the underlying static versus contextual model architecture.

**Figure 1.**
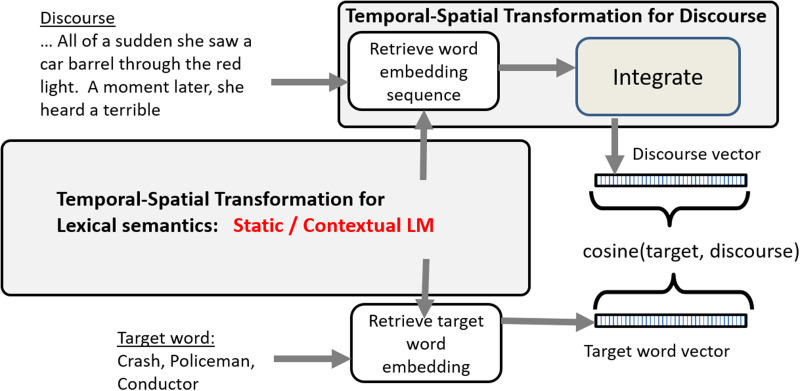
Model evaluation pipeline. For static models, separate embeddings are collected for each word in the discourse and integrated as an average vector to generate the discourse vector. In related studies this integration is performed by a recurrent reservoir model. For contextual language models (LMs), the language model generates the contextual discourse vector directly based on the input discourse. In parallel, the embedding is retrieved for the target word. The embeddings for the discourse and the target word are compared by the cosine of the angle between them, with 1 as identical and 0 as orthogonal.

We compared inference performance and semantic performances for 22 static LMs based on word2vec and GloVe and for 23 contextual LMs based on BERT/RoBERTa. These are listed in Appendix 1 in the Supporting Information, which is available at https://doi.org/10.1162/nol_a_00090. The processing pipeline for evaluating a model is illustrated in Figure 1.

In the inference tasks, the given text is run through the LM. For the static models, embeddings are produced for each word and accumulated as an average vector. For the contextual models the integration is performed by the model, and we retrieve the contextual embedding. For this we used the sentence transformer adaptation of BERT and RoBERTa (Liu et al., 2019)—SBERT (Reimers & Gurevych, 2019). SBERT adds a pooling operation to the output of BERT/RoBERTa to derive a fixed sized sentence embedding that renders BERT capable of producing semantically meaningful sentence embeddings. The SBERT.net infrastructure provides access to a number of the pretrained BERT and RoBERTa models that we used. Pretraining corpora include the semantic textual similarity baseline (STSb) corpus (Cer et al., 2017) and the Stanford natural language inference (SNLI) corpus (Bowman et al., 2015).

We then calculated the distance between the text embedding and the target word in the inference task using the cosine similarity, which is a measure of the vector similarity. This is explained in more detail for each task below, including the linking hypotheses between the cosine similarity measure and the human performance measures.

### Semantic and Inference Tasks

In this study, we adopted two inference tasks based on hallmark studies of Metusalem (Metusalem et al., 2012) and McKoon (McKoon & Ratcliff, 1986). In order to compare inference performance with more basic semantic processing baselines, we also adopted two measures of semantic processing from Chwilla et al. (1995), and a measure derived from the Metusalem study.

#### Chwilla semantic task

In this task (Chwilla et al., 1995), subjects were exposed to a priming word and then a target word, and the N400 in response to the target word was measured. Chwilla et al. provide 40 pairs of related words and 40 pairs of unrelated words. In a lexical decision task, targets that were unrelated to the prime produced larger N400 responses than related targets. In the linking hypothesis for this measure, our measure of cosine similarity is inversely related to the N400 in the Chwilla task. For our model testing, the Chwilla semantic score was computed by the following formula.

$$\text{Chwilla}\;\text{Semantics}=\text{CosSim}\left(\text{Related}\;\text{pair}\right)-\text{CosSim}\left(\text{Unrelated}\;\text{pair}\right)$$ (1)

To evaluate the performance of a given LM model, we compared the semantic relatedness predicted by the model for the related versus unrelated pairs. We then performed a *t* test on the semantic relatedness scores for the 40 related versus 40 unrelated pairs. The *t* score of the comparison is the indicator of performance. A significant result corresponds to a modeling of the human N400 behavior according to the linking hypothesis stated above.

#### Metusalem semantic task

In order to obtain an alternative measure of semantic processing, we used the Metusalem task and compared responses to the expected versus unexpected (related and unrelated) words. We generated an average vector for the words in the sentence context (see Table 1), and then compared cosine similarity between sentence and the expected word versus the mean of the similarities of the sentence to the two unexpected words. This difference is a measure of semantic processing. In the linking hypothesis for this measure, our measure of cosine similarity is inversely related to the N400 that would be elicited under these conditions. The score of semantics based on the Metusalem study (Metusalem et al., 2012) was computed by the following formula.

MetusalemSemantics=CosSim(SentVect,Expected)−(CosSim(SentVect,Related)+CosSim(SentVect,Unrelated))/2 (2)

We performed a paired *t* test on the semantic relatedness scores for the expected versus unexpected stimuli in the 72 experimental trials provided by Metusalem et al. The *t* value of the comparison is the measure of performance.

#### Metusalem inference task

In the Metusalem inference task (Metusalem et al., 2012), the N400 was measured in response to the expected, related, and unrelated words. The response to the related words decreases in the discourse vs. sentence context. In the current study, we used a measure of cosine similarity between sentence/discourse vectors and word vectors. This measure increases as these vectors are more similar. In the linking hypothesis for this measure, our measure of cosine similarity was inversely related to the N400 in the Metusalem task. Thus, the score of inference based on the Metusalem task was calculated as follows:

MetusalemInference=CosSim(Context,Related)−CosSim(Context,Unrelated)−(CosSim(Sentence,Related)−CosSim(Sentence,Unrelated)) (3)

where Context is the vector representation of the discourse context along with the sentence, and Sentence is the vector representation of the sentence out of context (see Table 1). In other words, we measured the advantage of the complete discourse for differentiating between Related words versus Unrelated words (second line in Equation 3), with respect to the single sentence in differentiating between the Related and Unrelated (third line in Equation 3). The idea is that similarity to the Related words will be “rescued” by the extended discourse, and not by the sentence alone.

**Figure 2.**
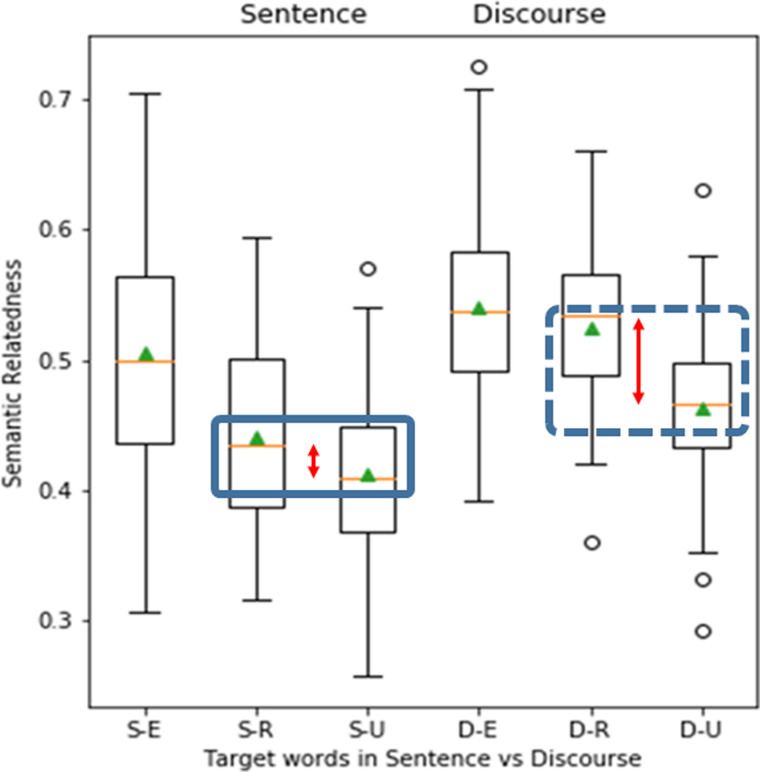
Example results of Metusalem inference for a single static model using the enwiki_20180420_100d corpus. The measure of inference is indicated in the solid and dotted horizontal boxes. In the Sentence condition, there is a small difference between S-R and S-U (solid box). When discourse context is provided, the relative similarity for the related words (D-R) is rescued (dotted box). Inference is measured by the difference between discourse vs. sentence (DR-DU) − (SR-SU), as specified in Equation 3. The red arrows indicate the rescue as the semantic relatedness advantage for related vs. unrelated words is increased in the discourse context. S = Sentence, D = Discourse context. E = Expected, R = Unexpected Related, U = Unexpected Unrelated.

To evaluate a model, we ran the model on the sequence of words in the sentence or the extended context discourse to generate the sentence/context vector. We then generated the embedding vector for the target word and measured the cosine similarity between the target word and sentence/context vector. We performed a paired *t* test on the semantic relatedness scores for Context(Related-Unrelated) versus Sentence(Unexpected-Related) for the 72 experimental trials provided by the Metusalem et al. The *t* value of the comparison is the measure of performance. An evaluation of the static wikipedia2vec model enwiki_2018042_100d on the 72 trials is presented in Figure 2. There we see indeed, that the discourse context rescues the similarity for the Related word. That is, the advantage of Unexpected-Related over Unexpected-Unrelated is revealed in the Discourse context. The comparison in Equation 3 yields *t* statistic = 4.14, *p* = 9.3e−05.

#### McKoon inference task

In the McKoon task (McKoon & Ratcliff, 1986), subjects displayed greater reaction times in response to a word when it was evaluated in a matching inference context sentence against a control sentence made with most of the same words but not evoking the event context. We created a linking hypothesis, such that the value that we calculate, based on cosine similarity, varies inversely with the reaction time in the original McKoon task. In our study, the score for evaluating performance on this task was calculated as follows:

$$\text{McKoon}\;\text{Inference}=\text{CosSim}\left(\text{Context},\text{Target}\right)-\text{CosSim}\left(\text{Control},\text{Target}\right)$$ (4)

To evaluate a model, the model was run on the context sentence and the control sentence, and the resulting vectors were compared to the target word vector, for the 32 experimental trials provided in McKoon and Ratcliff (1986). We performed a paired *t* test on these semantic relatedness values. The *t* value of the comparison is the measure of performance.

## RESULTS

Globally, we tested 22 static LMs and 23 contextual LMs on 2 semantic tasks, and 2 inference tasks. Model details are provided in the Supporting Information. For the semantic tasks, all models successfully performed the Chwilla semantic task, and all but one static model (Static LM 11; see Appendix in Supporting Information) and one contextual model (Contextual LM 10) successfully performed the Metusalem semantic task. For inference, all but three of the static models (Static LMs 11, 18, 19) successfully performed Metusalem, and all but three static models (Static LMs 2, 12, 18) successfully performed the McKoon inference task. Successful performance was characterized by *p* < 0.05 on the *t* statistics described for Equations 1–4. Note that while we mention significance here, in the main analyses that follows we use these *t* values in correlation analyses and do not interpret them in terms of significance. Thus we are not concerned with the possibility of false positives in these multiple comparisons.

In this section, we provide a brief roadmap for the four experiments we performed. In the first experiment we examined the correlation between semantics and inference for the two types of LM. This yielded a dissociation between the two forms of inference for both LMs, as semantics and inference were correlated for Metusalem but not McKoon inference. In Experiment 2, we performed the same comparisons, but this time using a measure of semantics that takes into account contextual processing. This further clarified the dissociation between the two forms of inference, such that the correlation between semantics and inference was rescued for McKoon inference only for the contextual LMs. In Experiment 3 we directly contrasted performance on the inference tasks for the two LMs, which clarified that McKoon inference requires contextual processing. Experiment 4 performed a complementary analysis, this time distinguishing between semantic tasks that have different requirements on integrative processing.

### Experiment 1

The objective of Experiment 1 was to test the prediction that for a given LM, performance in a semantic task should predict or be correlated with performance in an inference task. For semantics we used the Chwilla task, and for inference we used the Metusalem and McKoon tasks. We tested the correlations between semantic and inference performance using two populations of LMs: static and contextual.

**Figure 3.**
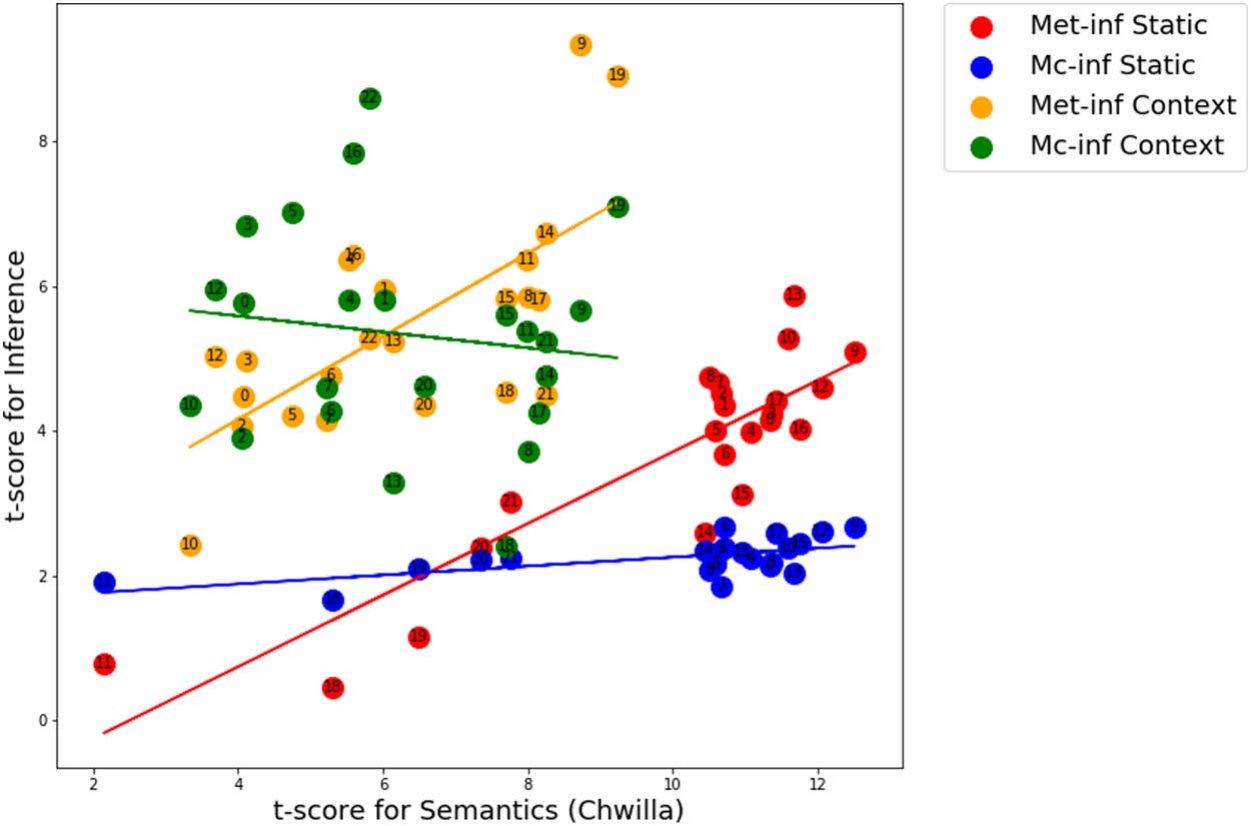
Experiment 1. Relation between Chwilla semantics and Metusalem/McKoon inference by static and contextual LMs. Both classes of models display significant correlation between semantic and inference for the Metusalem task (red and orange lines). Interestingly, this is not the case for the McKoon task, where both fail to demonstrate such effective correlation between semantic and inference performance (blue and green lines). Each data point in the scatterplot refers to a specific LM, and the number corresponds to the numbered LMs (see Supporting Information for static and contextual LMs). Met-inf = Metusalem et al. (2012) inference, Mc-inf = McKoon and Ratcliff (1986) inference.

Thus, for each of the static and contextual models, we tested the model on the semantic task and the two inference tasks, and we compared performance on semantics vs. inference for the two inference tasks. Figure 3 shows the result of comparison between Chwilla semantics and Metusalem and McKoon inference for static and contextual LMs. We observed a correlation between performance for semantic and inference processing for Metusalem inference, such that increased performance on semantics corresponds to increased performance on inference. This is the case for both for the static (Pearson correlation *r* = 0.88, *p* = 7.16e−08; regression slope 0.49) and contextual (*r* = 0.68, *p* = 0.0004; regression slope 0.57) LMs. In contrast, for McKoon inference, increased performance on semantics is not associated with increased performance in inference, neither for the static nor the contextual LMs. For the static model, while the correlation is significant (Pearson *r* = 0.60, *p* = 0.003), the slope of the regression line is nearly flat, at 0.06. This low slope indicates that as performance improves with semantics, it changes marginally for inference. Likewise, for the contextual LM, the correlation is not significant (*r* = −0.13, *p* = 0.5) and the slope of the regression is −0.11.

These results demonstrate a dissociation between inference processing as evaluated by the Metusalem and McKoon tasks when compared with semantic processing as evaluated by the Chwilla task. This is observed both for static and contextual LMs. It is of interest to examine the variability across the different model groups. For example, for Metusalem inference the best two contextual models, 9 and 19 both correspond to versions that employ the MPNet training method (Song et al., 2020; see Supporting Information). This shows a coherence across these model types with respect to performance on these tasks.

### Experiment 2

Experiment 1 revealed that the correlation between classes of language processing tasks—semantics versus inference—varies depending on the specific inference task. This suggests that there is some non-trivial difference between the two inference tasks. However, it is also possible that the semantic task plays a role in this dissociation. We thus set out in Experiment 2 to examine the correlation between semantic and inference processing using a different semantic task. We used a measure of semantics that exploits a dimension of the Metusalem task that compares expected versus unexpected words in the context of a target sentence. This semantic task is thus more elaborate than a simple difference between related versus unrelated word pairs. We compared this measure of Metusalem semantics and Metusalem/McKoon inference for the static and contextual LMs, as in Experiment 1.

**Figure 4.**
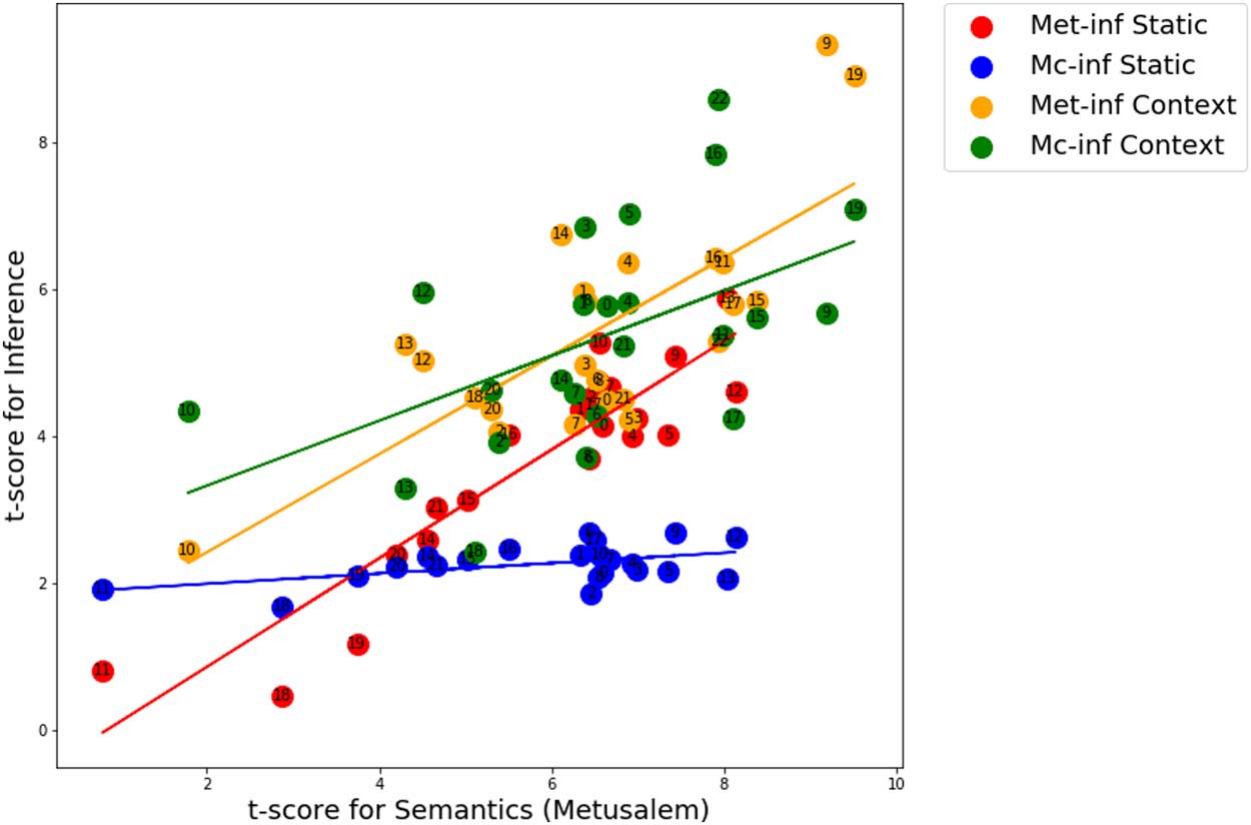
Experiment 2. Relation between Metusalem semantics and Metusalem/McKoon inference with Static and Contextual LMs. Contextual models now display significant correlations between semantic and inference processing for both inference tasks. Only the static models with the McKoon task fail to display clear effective correlation with semantics. Each data point in the scatterplot refers to a specific LM, and the number corresponds to the numbered LMs (see the Supporting Information for static and contextual LMs).

Figure 4 illustrates the result of comparison between Metusalem semantics and Metusalem/McKoon inference for static and contextual LMs. Similar to Experiment 1, for the static models, we continued to observe a clear positive relation between semantics and inference for Metusalem, but not for McKoon. Interestingly, with the contextual models the semantics-inference relation is rescued for McKoon inference. That is, with the contextual LMs, when the more elaborate measure of semantics is used the correlation between inference and semantics is recovered for McKoon inference (regression slope 0.44, Pearson *r* = 0.51, *p* = 0.01). This is not the case for the static models (regression slope 0.07, Pearson *r* = 0.48, *p* = 0.02). For Metusalem inference, there is a clear relation between semantics and inference for the static (regression slope 0.74, Pearson *r* = 0.91, *p* = 2.6e−9) and contextual LMs (regression slope 0.67, Pearson *r* = 0.75, *p* = 3.3e−5). This reveals complex interactions between LM classes, inference types, and semantic types.

### Experiment 3

**Figure 5.**
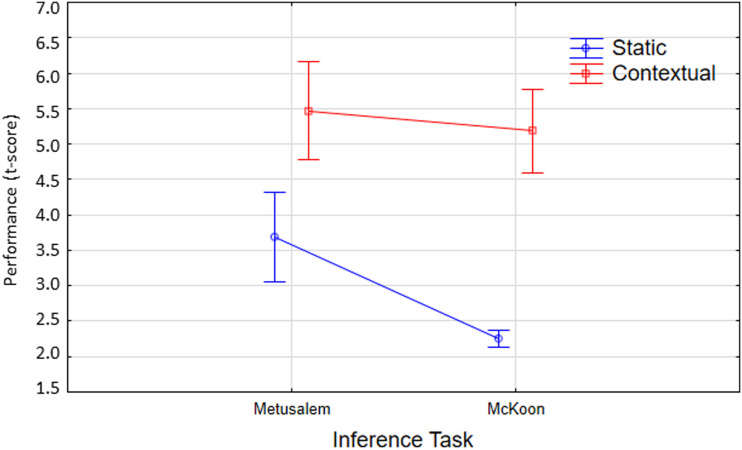
Mean performance for static and contextual models on the Metusalem and McKoon inference tasks (as characterized by the *t* scores on comparisons). The contextual models perform well on both tasks, while the static models perform better on Metusalem vs. McKoon. Whiskers denote 0.95 confidence intervals.

In order to gain a more direct view of the relation between Metusalem and McKoon inference, we compared them directly, again for the static and contextual models. We observed that static models displayed increased performance for Metusalem inference, relative to their reduced performance on McKoon inference (Figure 5). In contrast, contextual models displayed good performance both for Metusalem and McKoon inference and outperformed the static models on both inference tasks.

These observations are confirmed by a 2 × 2 analysis of variance (ANOVA) on Inference Task (Metusalem, McKoon) × Model (static, contextual). There is a significant main effect for Inference Task, with Metusalem having higher scores than McKoon (main effect for Task *F*(1, 21) = 18, *p* < 0.001). There is a significant main effect for Model, as contextual models perform better than the static models (main effect for Model *F*(1, 21) = 55, *p* < 0.001). While contextual models performed equally well for both tasks (Scheffe post hoc *p* > 0.1), the static models performed significantly better on Metusalem than McKoon inference (Scheffe post hoc *p* < 0.001). Task-related differences for the models are revealed in the significant Task × Model interaction (*F*(1, 21) = 5.5, *p* < 0.05).

These performance differences can be considered in terms of the nature of the tasks and the models. Both inference tasks involve processing of multiple word sentences, and so there may be an overall advantage for the contextual models. The Metusalem task involves unambiguous interpretation of multiple target words, whereas the McKoon task involves multiple interpretations of the same word in different contexts. By construction, the requirement in McKoon inference for contextual sensitivity favors Contextual models and can help us to understand the observed Task × Model interaction.

### Experiment 4

**Figure 6.**
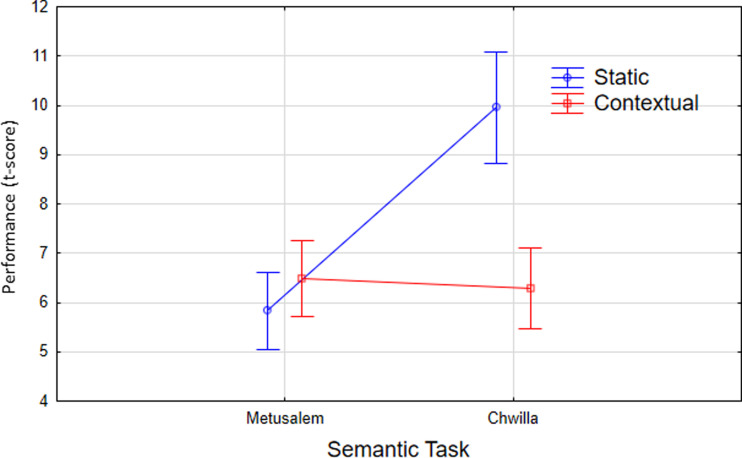
Experiment 4. Comparison between performance (as characterized by the *t* scores on comparisons) on the two semantic tasks for the static and contextual LMs. While contextual models dominated for inference processing (see Figure 5), the best scores for the Chwilla semantic task are attained by the static models.

As we observed significant task-related difference for the inference tasks, we completed the analysis by directly comparing our two semantic tasks in the context of the two LM classes. Figure 6 illustrates the comparisons between performance on the two semantic tasks for the static and contextual models. Performance is characterized by the *t* statistic value for the models on the inference tasks. Interestingly, we observed that the static models displayed superior performance for the Chwilla versus Metusalem semantic tasks, and indeed that on the Chwilla task, the static models actually outperformed the contextual models.

These observations are confirmed by the 2 × 2 ANOVA on Model (static, contextual). × Semantic Task (Metusalem, Chwilla). The static models outperformed the contextual models (main effect for Model *F*(1, 21) = 5.1, *p* < 0.05). The Chwilla task yielded superior overall performance (main effect for Semantic Task *F*(1, 21) = 114; *p* < 0.001). The significant performance increase specifically for the static model with Chwilla semantics is confirmed by the significant Model × Task interaction (*F*(1, 21) = 86, *p* < 0.001).

This advantage for static models on the Chwilla task might be understood in terms of the behavioral requirements of the semantic tasks and the nature of the static and contextual LMs. The Chwilla semantic task is based on evaluating relations between single word pairs. This corresponds to the word centered representations in the static models, thus providing a potential advantage for the static models.

## DISCUSSION

The current research explores how event knowledge that is not explicitly stated in the narrative may be coded in LMs, and how this can be used to better understand the computational neurophysiology of human inference processing. This research takes place in the context of an exciting new development in human language neurophysiology, that exploits a form of “big science” based on massive availability of data sets and multiple models (e.g., Schrimpf et al., 2021). Within this context, the current research takes a complementary approach, that focuses on specific tasks within the same domain (inference) and the differences between them.

We focused on two well-characterized inference tasks from the human experimental literature. The Metusalem task examines how the addition of contextual discourse allows inferences about events to be made. In the behavioral procedure, the experimental variable measured was an implicit brain response to particular target words that were related to the discourse (or not) via inference. Thus, there was no explicit inference task. After each trial the participant was asked a simple Yes/No comprehension question to ensure that they read each scenario for comprehension.

The McKoon task examines how the reorganization of the same words in different sentences can change the event context to allow inferences about events. In contrast to the Metusalem task, in the McKoon task, the experimental variable measured was an explicit behavior (response time) to a question that required inference on the event described in the sentence. These explicit questions encourage the subjects to make more strategic and deeper encodings of the inferred event.

In the classic context of inferencing, as characterized in the cited works of Metusalem and McKoon, at least some forms of inference are assumed to rely on rather elaborate cognitive processes that include the construction of situation models that represent agents, actions, causal roles, and so on (Zwaan, 2014; Zwaan et al., 1995). However, McKoon and Ratcliff (1992) argue for a minimalist hypothesis of inference, where automatic inference during reading relies only on easily available information, while more elaborate representations are constructed by dissociable processes, as required for specific, strategic needs. We can interpret our current results in this context, where the Metusalem inferencing task relies preferentially on this minimalist implicit processing, which can be furnished by static LMs. In contrast, the McKoon inference task requires representations associated with more explicit strategic processing and that are more associated with the contextual LMs.

In other words, the McKoon inference task is sensitive to how the contextual organization of words in a sentence can change its relation to a single target word. The target sentence evokes the test word by inference. The control sentence uses as many of the same words as possible, but organized in a different order, and does not evoke the test word. This requirement for disambiguation is consistent with contextual LMs. On the other hand, the Metusalem inference task is sensitive to how the simple accumulation of word information in a text can disambiguate a related word by inference, but it is not specifically sensitive to the contextual organization of words within a sentence or discourse. This can be achieved using aggregation of word embeddings from a static LM. In summary, static LMs are able to simulate minimalist inference processing as required by the Metusalem task, whereas contextual LMs are required to simulate the more strategic processing as required for the McKoon task. It is noteworthy that we specifically chose not to use “left-to-right” autoregressive models/transformers (e.g., GPT-2). This is because we wanted to take an Occam’s razor approach, using the simplest models that can reveal the distinction between implicit and explicit inference processing. This said, autoregressive models can be seen as interesting from the psycholinguistics point of view, and testing such models would make sense in a future avenue of research.

We have identified two forms of inference processing (Metusalem and McKoon) that are realized by human neural mechanisms that have not yet been fully characterized. For these inference processes, we defined linking hypotheses between the observed human behavior or neurophysiology and the response of the LM. In this context we have demonstrated that these two forms of inference rely on dissociable computational processes corresponding to static and contextual LMs. This allows us to propose that these two forms of inference should also rely on dissociable neural mechanisms.

This functional distinction should now translate into patterns in language processing in the human brain that we would predict based on our results. In this context, Schrimpf et al. (2021) characterized the brain score of LMs as a function of their ability to predict human neural data recorded while subjects perform language comprehension (typically self-paced reading). They observed that brain score is correlated with the ability to predict the next word in text but not necessarily with the ability to perform other language tasks, including inferencing (evaluated as a subset of language tasks in Wang et al., 2018).

Interestingly, the brain activity used in these analyses was recorded while subjects were reading but not directly performing other language tasks. This leads us to propose the hypothesis that if neural activity is recorded while subjects are performing tasks that require inference processing, then brain scores on such a data set will correlate with the performance on the corresponding inference tasks. Indeed, it is highly likely that behaviorally dissociable forms of language processing will be associated with distinct neural computations and distinct spatiotemporal patterns of brain activation.

More specifically, we predict that when human subjects are exposed to comprehension tasks that have been computationally dissociated, as with the Metusalem and McKoon inference tasks, this processing will be accompanied by dissociable spatiotemporal patterns of brain activity.

We can consider that there are minimalist and more extended forms of inference as demonstrated by McKoon and Ratcliff (1992). Metusalem inference can rely on a shallow form of inference that is modeled by the accumulation of static LM embeddings. In contrast, McKoon inference is more appropriately modeled by contextual LMs. We thus predict that when humans are submitted to inference tasks, we should see neurophysiological evidence for these dissociable forms of inference. Specifically, performing a systematic search should identify regions of interest (ROIs) or networks whose activation is correlated with the shallow embedding accumulation as solicited by the Metusalem task, and another set of ROIs whose activation is correlated with the deeper transformer-based representations as solicited by the McKoon task.

It will be of particular interest to identify the brain networks that are solicited in inference tasks that require more explicit strategic processing and to compare these with networks that have been associated with automatic semantic processing. Binder and colleagues (Binder & Desai, 2011; Binder et al., 2009) have performed extensive meta-analysis of studies that involve semantic memory. They conclude that in addition to modality specific sensory-motor areas, semantic memory recruits high-level convergence zones in the inferior parietal, lateral temporal, and ventral temporal cortex. They note that these regions are far from primary sensory and motor cortices and appear to be involved in processing general rather than modality-specific semantic information (Binder & Desai, 2011). A related study likewise observed a corresponding distributed system for comprehension of pictures and sentences depicting human events (Jouen et al., 2015). Interestingly, the task in this experiment was simply to remain vigilant and respond to occasional questions that asked about what was shown in the previous stimulus. Despite the low requirements for explicit strategic processing, activation of an extensive semantic network was observed.

Future research should examine how task requirements, such as the need to perform strategic inference as required for the McKoon task, will recruit brain networks not seen for more automatic processing. The specification of computationally dissociable instances of the same class of tasks, as we have done here for Metusalem and McKoon inference, provides the basis for discovering whether these computational dissociations are reflected in distinct neural computations in the human brain. It is likely that this progress will inform and be informed by the cognitive processing of events (e.g., Radvansky & Zacks, 2017). Indeed, mounting neuroscientific evidence shows how event structure is processed in the brain in terms of the spatial and temporal distributions of narrative event structure (Baldassano et al., 2017, 2018), and recurrent models of cortical processing are beginning to provide neurocomputational explanations for these event-driven phenomena (Dominey, 2021). What remains to further explore is the link between the computational process, such as those we explore here, and the spatiotemporal distribution of neural activity observed in the brain during narrative comprehension that relies on inference.

## FUNDING INFORMATION

Peter Ford Dominey, Conseil régional de Bourgogne-Franche-Comté (https://dx.doi.org/10.13039/501100011773), Award ID: RobotSelf. Hiroshi Ishiguro, Moonshot Research and Development Program (https://dx.doi.org/10.13039/501100020963), Award ID: JPMJMS2011. Hiroshi Ishiguro, Japan Society for the Promotion of Science (JSPS) Kakenhi grant, Award ID: 19H05693. Hiroshi Ishiguro, JSPS Kakenhi grant, Award ID: 22K17949.

## AUTHOR CONTRIBUTIONS

**Takahisa Uchida**: Conceptualization: Equal; Formal analysis: Lead; Investigation: Equal; Writing – original draft: Lead; Writing – review & editing: Equal. **Nicolas Lair**: Conceptualization: Supporting; Methodology: Supporting; Writing – review & editing: Supporting. **Hiroshi Ishiguro**: Funding acquisition: Lead; Resources: Lead; Supervision: Supporting; Writing – review & editing: Supporting. **Peter Ford Dominey**: Conceptualization: Equal; Formal analysis: Supporting; Funding acquisition: Equal; Project administration: Lead; Writing – original draft: Supporting; Writing – review & editing: Equal.

## DATA AVAILABILITY STATEMENT

Analysis code and data for these experiments are openly available at this repository: https://github.com/TakahisaUchida/InferenceSemanticsExperiment.

## Supplementary Material



## TECHNICAL TERMS

Language model: Captures salient statistical characteristics of word distributions in a natural language corpus and allows representations of words as vectors (embeddings) that have useful semantic computational properties.
Embedding: Real-valued vector, generated by a language model, that encodes word meanings with useful properties; for example, words that are closer in the vector space tend to have related meanings.
Cosine similarity: Cosine of the angle between two word embedding vectors using the dot product, which is 1 for parallel vectors and 0 for tangent vectors, calculated to compare their semantic similarity.
