# Supplementary material for: Dissociable Neural Mechanisms for Human Inference Processing Predicted by Static and Contextual Language Models
Source: Neurobiol Lang (Camb). 2024 Apr 1;5(1):248–63. doi: 10.1162/nol_a_00090 (PMC11025649; doi:10.1162/nol_a_00090)
Supplement: Supplementary file 1 [file nol-5-1-248-s001.pdf]

Supplementary material Appendices:

A1. **Static LMs - Word2vec and GloVe:** The following 22 trained model of word2vec/GloVe word2vec/GloVe are used for our experiments. References for embedding algorithms are provided below.

Corpus (abbreviation)	Corpus contents	Embedding algorithms	Embedding vector dimension
0. enwiki_20180420_100d	Wikipedia	Word2vec[1]	100
1. enwiki_20180420_300d	Wikipedia	Word2vec[1]	300
2. enwiki_20180420_500d	Wikipedia	Word2vec[1]	500
3. enwiki_20180420_nolg_100d	Wikipedia	Word2vec[1]	100
4. enwiki_20180420_nolg_300d	Wikipedia	Word2vec[1]	300
5. enwiki_20180420_nolg_500d	Wikipedia	Word2vec[1]	500
6. enwiki_20180420_win10_100d	Wikipedia	Word2vec[1]	100
7. enwiki_20180420_win10_300d	Wikipedia	Word2vec[1]	300
8. enwiki_20180420_win10_500d	Wikipedia	Word2vec[1]	500
9. en_core_web_lg	blogs, news, comments	tok2vec[6]	300
10. en_core_web_md	blogs, news, comments	tok2vec[6]	300
11. en_core_web_sm	blogs, news, comments	tok2vec[6]	96
12. GENSIM-fasttext-wiki-news-subwords-300	Wikipedia 2017, UMBC webbase corpus and statmt.org news	Fasttext[2]	300
13. GENSIM-word2vec-google-news-300	Google News dataset	Word2vec[3]	300
14. GENSIM-glove-wiki-gigaword-50	Twitter	GloVe[4]	50
15. GENSIM-glove-wiki-gigaword-100	Twitter	GloVe[4]	100
16. GENSIM-glove-wiki-gigaword-200	Twitter	GloVe[4]	200
17. GENSIM-glove-wiki-gigaword-300	Twitter	GloVe[4]	300
18. GENSIM-glove-twitter-25	Twitter	GloVe[4]	25
19. GENSIM-glove-twitter-50	Twitter	GloVe[4]	50
20. GENSIM-glove-twitter-100	Twitter	GloVe[4]	100
21. GENSIM-glove-twitter-200	Twitter	GloVe[4]	200

A2. **Contextual LMs - BERT (Bidirectional Encoder Representations from Transformers):**

The following 23 pre-trained models are used for our experiments.

Corpus (abbreviation)	Corpus contents	Embedding algorithms	Embedding vector dimension
0. nli-bert-base	NLI	bert-base-uncased[5]	768
1. nli-bert-base-cls-pooling	NLI	bert-base-uncased[5]	768
2. nli-bert-base-max-pooling	NLI	bert-base-uncased[5]	768
3. nli-bert-large	NLI	bert-large-uncased[5]	1024
4. nli-bert-large-cls-pooling	NLI	bert-large-uncased[5]	1024
5. nli-bert-large-max-pooling	NLI	bert-large-uncased[5]	1024
6. nli-distilbert-base	NLI	distilbert-base-uncased[5]	768
7. nli-distilbert-base-max-pooling	NLI	distilbert-base-uncased[5]	768
8. nli-distilroberta-base-v2	NLI	distilroberta-base[5]	768
9. nli-mpnet-base-v2	NLI	mpnet-base[5]	768
10. nli-roberta-base	NLI	roberta-base[5]	768
11. nli-roberta-base-v2	NLI	roberta-base[5]	768
12. nli-roberta-large	NLI	roberta-large[5]	1024
13. paraphrase-distilroberta-base-v1	Paraphrase Data	distilroberta-base[5]	768
14. paraphrase-xlm-r-multilingual-v1	Paraphrase Data	XLM-R[5]	768
15. stsb-bert-base	bert-base-uncased	NLI+STSb[5]	768
16. stsb-bert-large	NLI+STSb	bert-large-uncased[5]	1024
17. stsb-distilbert-base	NLI+STSb	distilbert-base-uncased[5]	768
18. stsb-distilroberta-base-v2	NLI+STSb	distilroberta-base[5]	768
19. stsb-mpnet-base-v2	NLI+STSb	mpnet-base[5]	768
20. stsb-roberta-base	NLI+STSb	roberta-base[5]	768
21. stsb-roberta-base-v2	NLI+STSb	roberta-base[5]	768
22. stsb-roberta-large	NLI+STSb	roberta-base[5]	1024

[1] Yamada, Ikuya, et al. "Wikipedia2Vec: An efficient toolkit for learning and visualizing the embeddings of words and entities from Wikipedia." arXiv preprint arXiv:1812.06280 (2018).

[2] Mikolov, Tomas, et al. "Advances in pre-training distributed word representations." arXiv preprint arXiv:1712.09405 (2017).

[3] Tomas Mikolov, Kai Chen, Greg Corrado, and Jeffrey Dean. Efficient Estimation of Word Representations in Vector Space. In Proceedings of Workshop at ICLR, 2013.

[4] Pennington, Jeffrey, Richard Socher, and Christopher D. Manning. "Glove: Global vectors for word representation." Proceedings of the 2014 conference on empirical methods in natural language processing (EMNLP). 2014.

[5] Reimers, Nils, and Iryna Gurevych. "Sentence-bert: Sentence embeddings using siamese bert-networks." arXiv preprint arXiv:1908.10084 (2019).

[6] https://spacy.io/models/en

A3. Table of Inference/Semantic Score - Word2vec and GloVe

Corpus (abbreviation)	Metusalem Inference	McKoon Inference	Chwilla Semantics	Metusalem Semantics
0. enwiki_20180420_100d	t = 4.14, p = 9.32e-05	t = 2.14, p = 0.039	t = 11.34, p = 3.55e-18	t = 6.59, p = 6.43e-09
1. enwiki_20180420_300d	t = 4.35, p = 4.45e-05	t = 2.38, p = 0.023	t = 10.70, p = 5.60e-17	t = 6.33, p = 1.89e-08
2. enwiki_20180420_500d	t = 4.51, p = 2.44e-05	t = 1.85, p = 0.073	t = 10.67, p = 6.43e-17	t = 6.45, p = 1.17e-08
3. enwiki_20180420_nolg_100d	t = 4.23, p = 6.73e-05	t = 2.18, p = 0.036	t = 11.35, p = 3.35e-18	t = 6.99, p = 1.22e-09
4. enwiki_20180420_nolg_300d	t = 3.99, p = 0.00015	t = 2.24, p = 0.031	t = 11.06, p = 1.16e-17	t = 6.92, p = 1.59e-09
5. enwiki_20180420_nolg_500d	t = 4.01, p = 0.00014	t = 2.15, p = 0.038	t = 10.59, p = 9.16e-17	t = 7.35, p = 2.63e-10
6. enwiki_20180420_win10_100d	t = 3.68, p = 0.00044	t = 2.67, p = 0.011	t = 10.71, p = 5.54e-17	t = 6.42, p = 1.28e-08
7. enwiki_20180420_win10_300d	t = 4.65, p = 1.44e-05	t = 2.31, p = 0.027	t = 10.63, p = 7.71e-17	t = 6.67, p = 4.69e-09
8. enwiki_20180420_win10_500d	t = 4.74, p = 1.03e-05	t = 2.07, p = 0.046	t = 10.50, p = 1.33e-16	t = 6.54, p = 7.98e-09
9. en_core_web_lg	t = 5.08, p = 2.88e-06	t = 2.67, p = 0.011	t = 12.51, p = 2.47e-20	t = 7.42, p = 1.90e-10
10. en_core_web_md	t = 5.27, p = 1.39e-06	t = 2.37, p = 0.023	t = 11.59, p = 1.19e-18	t = 6.56, p = 7.42e-09
11. en_core_web_sm	t = 0.79, p = 0.42	t = -1.91, p = 0.065	t = 2.14, p = 0.035	t = -0.78, p = 0.43
12. GENSIM-fasttext-wiki-news-subwords-300	t = 4.60, p = 1.74e-05	t = 2.61, p = 0.013	t = 12.05, p = 1.67e-19	t = 8.13, p = 9.37e-12
13. GENSIM-word2vec-google-news-300	t = 5.86, p = 1.28e-07	t = 2.04, p = 0.049	t = 11.68, p = 8.26e-19	t = 8.03, p = 1.44e-11
14. GENSIM-glove-wiki-gigaword-50	t = 2.58, p = 0.011	t = 2.35, p = 0.025	t = 10.44, p = 1.79e-16	t = 4.56, p = 2.05e-05
15. GENSIM-glove-wiki-gigaword-100	t = 3.13, p = 0.0025	t = 2.32, p = 0.027	t = 10.95, p = 1.89e-17	t = 5.02, p = 3.63e-06
16. GENSIM-glove-wiki-gigaword-200	t = 4.02, p = 0.00014	t = 2.45, p = 0.019	t = 11.75, p = 5.96e-19	t = 5.50, p = 5.50e-07
17. GENSIM-glove-wiki-gigaword-300	t = 4.41, p = 3.51e-05	t = 2.58, p = 0.014	t = 11.42, p = 2.44e-18	t = 6.49, p = 9.60e-09
18. GENSIM-glove-twitter-25	t = 0.46, p = 0.64	t = 1.67, p = 0.10	t = 5.29, p = 1.04e-06	t = 2.87, p = 0.0053
19. GENSIM-glove-twitter-50	t = 1.16, p = 0.24	t = 2.08, p = 0.044	t = 6.49, p = 7.13e-09	t = 3.74, p = 0.00036
20. GENSIM-glove-twitter-100	t = 2.37, p = 0.02	t = 2.21, p = 0.034	t = 7.35, p = 1.68e-10	t = 4.19, p = 7.71e-05
21. GENSIM-glove-twitter-200	t = 3.02, p = 0.0034	t = 2.23, p = 0.032	t = 7.75, p = 2.78e-11	t = 4.66, p = 1.43e-05

A4. Table of Inference/Semantic Score - BERT

Corpus (abbreviation)	Metusalem Inference	McKoon Inference	Chwilla Semantics	Metusalem Semantics
0. nli-bert-base	t = 4.47, p = 2.86e-05	t = 5.77, p = 2.34e-06	t = 4.07, p = 0.00010	t = 6.63, p = 5.33e-09
1. nli-bert-base-cls-pooling	t = 5.95, p = 8.97e-08	t = 5.80, p = 2.16e-06	t = 6.02, p = 5.21e-08	t = 6.36, p = 1.68e-08
2. nli-bert-base-max-pooling	t = 4.06, p = 0.00012	t = 3.91, p = 0.00046	t = 4.04, p = 0.00012	t = 5.38, p = 8.96e-07
3. nli-bert-large	t = 4.95, p = 4.64e-06	t = 6.83, p = 1.16e-07	t = 4.11, p = 9.47e-05	t = 6.38, p = 1.54e-08
4. nli-bert-large-cls-pooling	t = 6.35, p = 1.72e-08	t = 5.80, p = 2.13e-06	t = 5.53, p = 4.04e-07	t = 6.87, p = 1.96e-09
5. nli-bert-large-max-pooling	t = 4.21, p = 7.26e-05	t = 7.01, p = 7.04e-08	t = 4.73, p = 9.46e-06	t = 6.89, p = 1.85e-09
6. nli-distilbert-base	t = 4.75, p = 1.0062e-05	t = 4.26, p = 0.00017	t = 5.27, p = 1.16e-06	t = 6.51, p = 8.83e-09
7. nli-distilbert-base-max-pooling	t = 4.15, p = 9.06e-05	t = 4.59, p = 6.90e-05	t = 5.21, p = 1.45e-06	t = 6.26, p = 2.57e-08
8. nli-distilroberta-base-v2	t = 5.84, p = 1.42e-07	t = 3.72, p = 0.00078	t = 8.00, p = 9.25e-12	t = 6.40, p = 1.40e-08
9. nli-mpnet-base-v2	t = 9.33, p = 5.73e-14	t = 5.67, p = 3.12e-06	t = 8.73, p = 3.55e-13	t = 9.18, p = 1.05e-13
10. nli-roberta-base	t = 2.43, p = 0.017	t = 4.34, p = 0.00013	t = 3.32, p = 0.0013	t = 1.77, p = 0.079
11. nli-roberta-base-v2	t = 6.36, p = 1.68e-08	t = 5.37, p = 7.32e-06	t = 7.99, p = 9.66e-12	t = 7.98, p = 1.77e-11
12. nli-roberta-large	t = 5.02, p = 3.57e-06	t = 5.95, p = 1.40e-06	t = 3.67, p = 0.00043	t = 4.50, p = 2.57e-05
13. paraphrase-distilroberta-base-v1	t = 5.24, p = 1.55e-06	t = 3.29, p = 0.0024	t = 6.13, p = 3.23e-08	t = 4.30, p = 5.20e-05
14. paraphrase-xlm-r-multilingual-v1	t = 6.73, p = 3.54e-09	t = 4.75, p = 4.27e-05	t = 8.24, p = 3.09e-12	t = 6.10, p = 4.84e-08
15. stsb-bert-base	t = 5.83, p = 1.49e-07	t = 5.60, p = 3.81e-06	t = 7.69, p = 3.61e-11	t = 8.38, p = 3.24e-12
16. stsb-bert-large	t = 6.42, p = 1.32e-08	t = 7.83, p = 7.63e-09	t = 5.59, p = 3.11e-07	t = 7.89, p = 2.66e-11
17. stsb-distilbert-base	t = 5.80, p = 1.69e-07	t = 4.24, p = 0.00018	t = 8.14, p = 4.83e-12	t = 8.10, p = 1.07e-11
18. stsb-distilroberta-base-v2	t = 4.52, p = 2.34e-05	t = 2.41, p = 0.021	t = 7.69, p = 3.66e-11	t = 5.11, p = 2.54e-06
19. stsb-mpnet-base-v2	t = 8.91, p = 3.43e-13	t = 7.08, p = 5.80e-08	t = 9.23, p = 3.73e-14	t = 9.51, p = 2.65e-14
20. stsb-roberta-base	t = 4.35, p = 4.44e-05	t = 4.61, p = 6.48e-05	t = 6.56, p = 5.24e-09	t = 5.30, p = 1.22e-06
21. stsb-roberta-base-v2	t = 4.49, p = 2.64e-05	t = 5.22, p = 1.12e-05	t = 8.25, p = 3.04e-12	t = 6.82, p = 2.42e-09
22. stsb-roberta-large	t = 5.28, p = 1.34e-06	t = 8.59, p = 1.05e-09	t = 5.80, p = 1.31e-07	t = 7.93, p = 2.19e-11
